# Decline in transmission of schistosomiasis mansoni in Oman

**DOI:** 10.1186/s40249-016-0210-1

**Published:** 2016-12-12

**Authors:** Idris Al Abaidani, Seif Al-Abri, Mahmoud Shaban, Satish L. Ghugey, Salem Al Kathery, Khalid Al-Mashikhi, Amadou Garba, Albis Francesco Gabrielli

**Affiliations:** 1 Directorate General for Disease Surveillance & Control, Ministry of Health, Muscat, Oman; 2 Undersecretary Office, Ministry of Health, Salalah, Oman; 3 Directorate General of Health Services, Dhofar Governorate, Ministry of Health, Salalah, Oman; 4 Department of Neglected Tropical Diseases, World Health Organization, Geneva, Switzerland; 5 Division of Communicable Diseases, World Health Organization Regional Office for the Eastern Mediterranean, Shara’a Abdul Razzak Al Sanhouri, PO Box 7608, Nasr City, 11371 Cairo Egypt

**Keywords:** Schistosomiasis, *Schistosoma mansoni*, Elimination, Parasitology, Serology, Oman

## Abstract

**Background:**

Intestinal schistosomiasis due to *Schistosoma mansoni* was first reported in Oman in 1979. We describe the trend in parasitological and serological prevalence of human infection with *S. mansoni* in the endemic area over the period 1982–2014, and the compliance of data generated by the national monitoring and evaluation system with schistosomiasis elimination criteria set by the Ministry of Health of Oman.

**Methods:**

Parasitological and serological assessments were carried out on population (mainly children) living in the area at risk for schistosomiasis in Dhofar, the country’s only endemic Governorate, for a period of over 30 years. Kato-Katz thick smear and Indirect Haemagglutination Assay were the techniques employed.

**Results:**

Data indicate a progressive decline in prevalence of *S. mansoni* throughout the 1980s and the 1990s, a recrudescence in the early 2000s, and a more marked decrease following the implementation of six rounds of mass treatment with praziquantel from 2007 to 2013. Latest parasitological prevalence (2011) was 0%, while latest serological prevalence (2014) was 0.11%.

**Conclusion:**

Transmission of schistosomiasis has reached very low levels in Oman. Elimination criteria established by the Ministry of Health of Oman (parasitological prevalence ≤ 1% and serological prevalence ≤ 5%) have been met since 2008. Further investigations are required to assess whether interruption of transmission has been achieved in some or all foci, in view of the establishment of a formal verification process under the auspices of WHO.

**Electronic supplementary material:**

The online version of this article (doi:10.1186/s40249-016-0210-1) contains supplementary material, which is available to authorized users.

## Multilingual abstracts

Please see Additional file 1 for translation of the abstract into six official working languages of the United Nations.

## Background

### Schistosomiasis and its elimination

Schistosomiasis is a blood-fluke (trematode) infection characterized by two major clinical presentations. *Schistosoma mansoni, S. mekongi, S. intercalatum* and *S. japonicum* are responsible for intestinal schistosomiasis, while *S. haematobium* causes urogenital schistosomiasis [1]. The burden of schistosomiasis is still significant in many parts of the world; however several countries have reached a low-endemic status [2]. Since the adoption of World Health Assembly Resolution WHA65.21 on “elimination of schistosomiasis” [3], and the release of the Schistosomiasis Strategic Plan 2012–2020 [2], WHO encourages low-burden countries to embark in the “final push” and cut transmission of schistosomiasis, thus achieving its elimination, through an inter-sectorial approach encompassing preventive chemotherapy, snail control, environmental management, health education, access to safe water, and sanitation.

As Oman is one of such countries, we aim to describe the trend in levels of parasitological and serological prevalence of human infection with *S. mansoni* over the period 1982–2014, and we discuss the compliance of data generated by the national monitoring and evaluation system with elimination criteria set by the Ministry of Health of Oman, as well as the steps required to further document interruption of *S. mansoni* transmission in the country, in view of the expected establishment of a formal verification process to be carried out under the auspices of WHO.

### Schistosomiasis in Oman

Both *Schistosoma mansoni* and *Schistosoma haematobium* are endemic in the Arabian peninsula, notably in Saudi Arabia and Yemen [4, 5].

In Oman, transmission of intestinal schistosomiasis due to *S. mansoni* has been documented only in Dhofar Governorate, and was first reported in 1979. *Biomphalaria arabica* (a strain of *B. pfeifferi*) has been identified as the snail intermediate host, and since 1896 has been observed in Dhofar, where its distribution is limited to few foci only. Extensive malacological investigations have shown that *B. arabica* is not present elsewhere in Oman and that no other *Biomphalaria* species is found in the country, [6–10] thus excluding the possibility of transmission of *S. mansoni* outside Dhofar.

Although the snail *Bulinus wrighti*, a potential intermediate host of *S. haematobium*, is present in Dhofar [10, 11] as well as in other limited areas of the country [12], transmission of *S. haematobium* has never been found to occur in Oman, and no autochthonous cases of urinary schistosomiasis have ever reported by the country [13, 14].

**Fig. 1:**
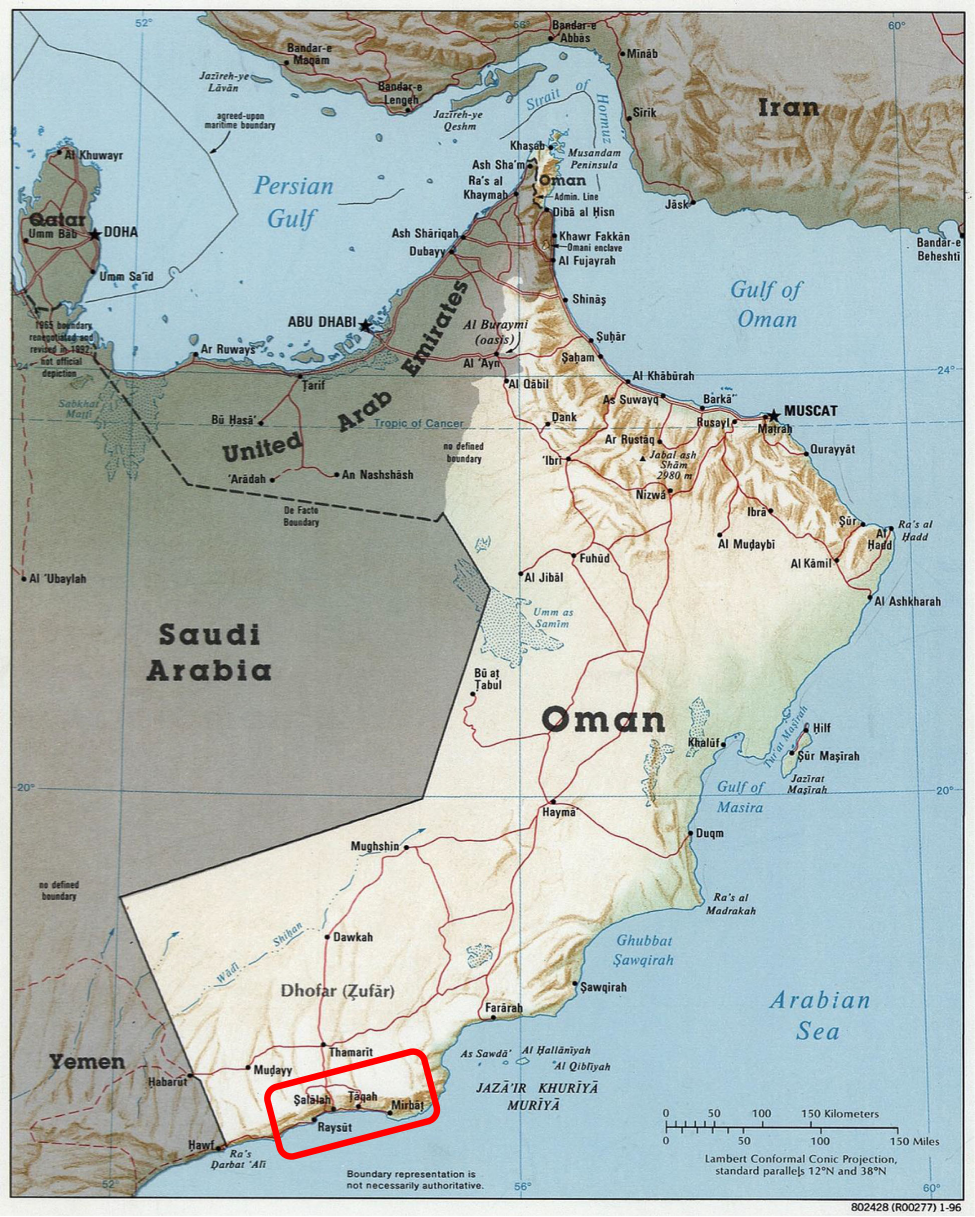
Map of Oman (in red: schistosomiasis endemic area in Dhofar Governorate)

**Fig. 2:**
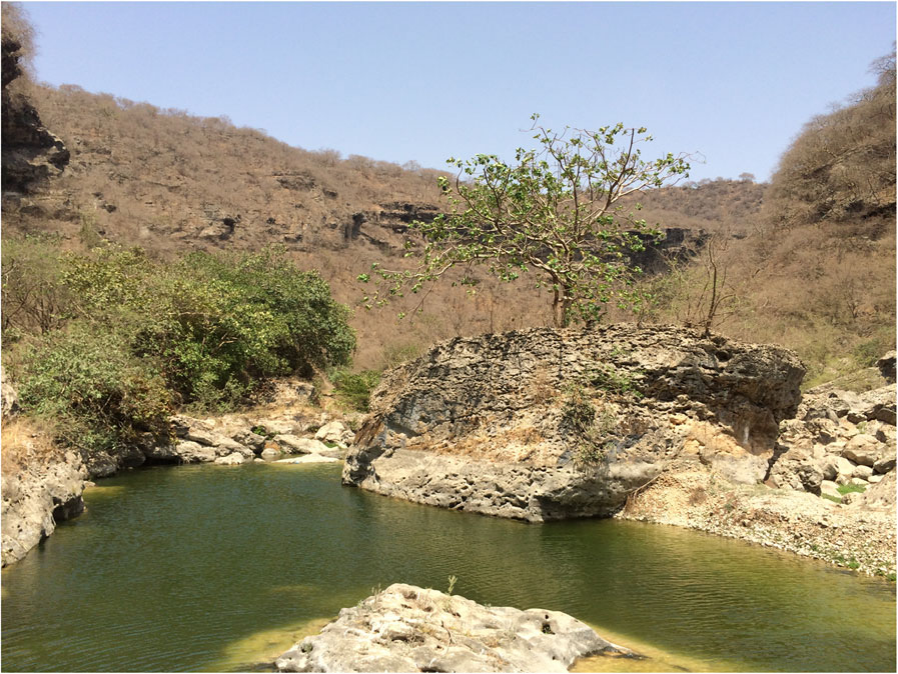
Natural water body in hilly Dhofar (WHO/AF Gabrielli)

**Fig. 3:**
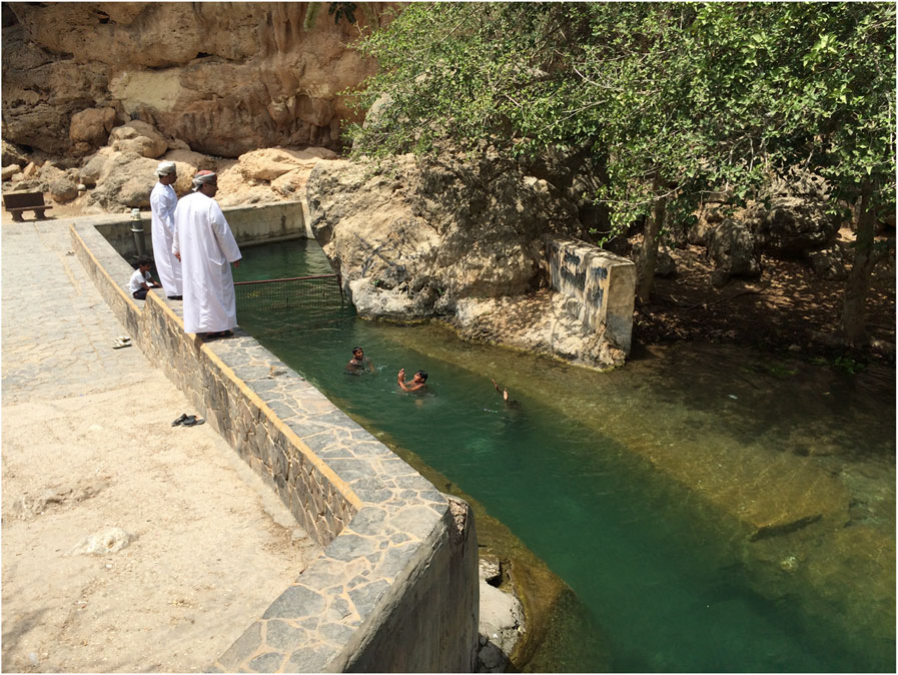
Artificial water body used for recreational purposes (WHO/AF Gabrielli)

Dhofar lies in south-western Oman, bordering Yemen. It is a rather mountainous region covering 99 300 sq km (38 300 sq mi) (Fig. 1), with a population of approximately 375 000. Dhofar’s weather is relatively cool and rainy even during the summer (July to September, e.g., in July, mean daily temperature = 26.4 °C, mean rainfall 24.5 mm). Schistosomiasis transmission sites include temporary and permanent, natural and artificial water bodies and riverbeds (wadis) located in a relatively small (80 km × 20 km), hilly area along the coast of the Indian Ocean [15] (Figs. 2 and 3). Such area lies within 20 km from the seashore, and is comprised in the *wilayat* (provinces) of Salalah, Taqah and Mirbat. Water contact used to be related to domestic activities but now mainly occurs for recreational purposes, although shepherds might bring their animals to water bodies, thus having occasional contacts. Human population in the area at risk for schistosomiasis is estimated at 25 000, including Omanis and non-Omanis.

In Dhofar, epidemiology of schistosomiasis has been monitored through an extensive active surveillance screening system. Passive surveillance is also in place; schistosomiasis is a notifiable disease under group B-Communicable Diseases, which means that suspect or confirmed cases detected through the routine health services of Oman have to be reported within a week time [16]. While a few cases of both *S. mansoni* and *S. haematobium* infection are still passively detected every year in several Governorates among non-Omanis and Omanis alike, thorough investigations have led to the conclusion that none, among the cases occurred in recent years, acquired the infection in Oman; as such they were all considered imported. The last passively-detected autochthonous case occurred in 2007: a case of intestinal schistosomiasis from Dhofar governorate [14].

### Schistosomiasis control in Dhofar

**Fig. 4:**
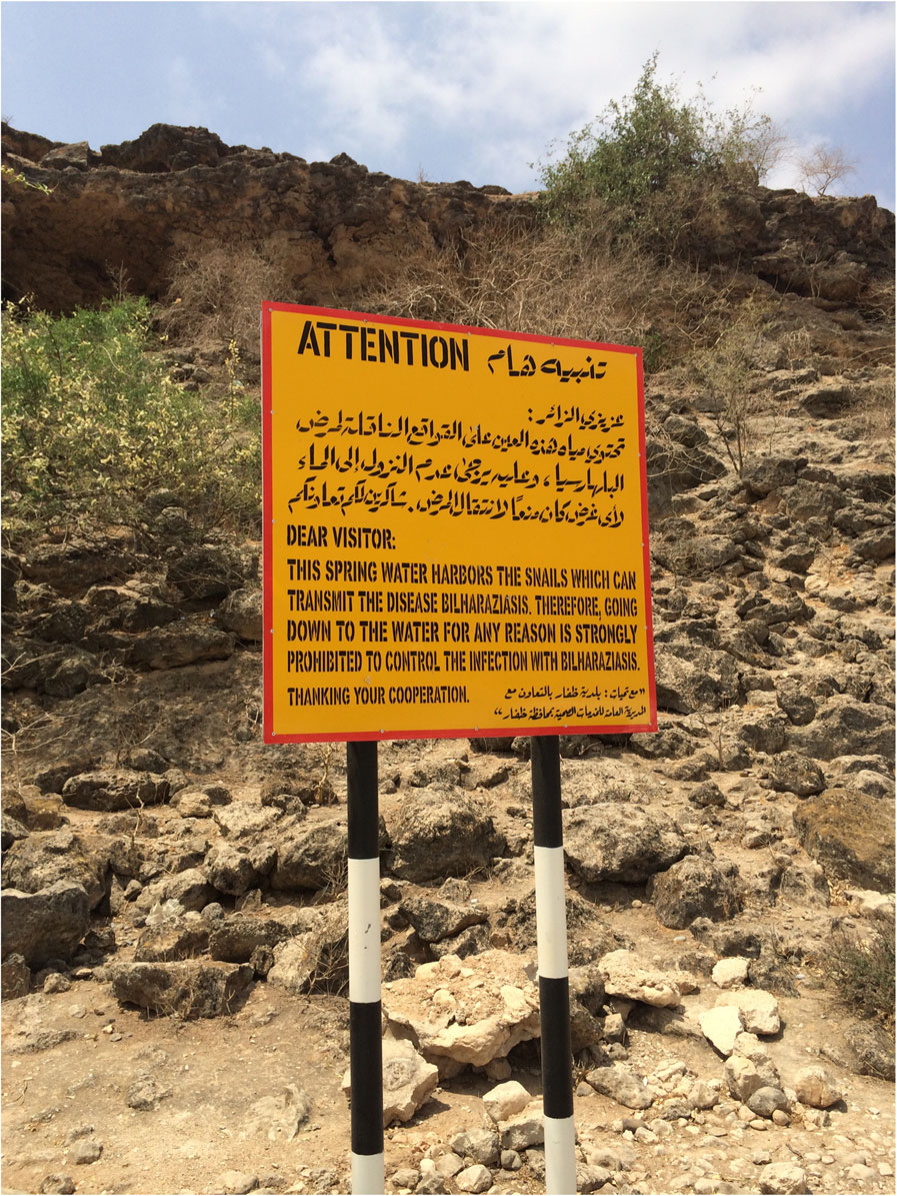
Sign advising not to enter water bodies (WHO/AF Gabrielli)

Epidemiological investigations on schistosomiasis, as well as planning of public-health measures to tackle the disease, followed the detection of the first two cases of schistosomiasis, at Sultan Qaboos Hospital in Salalah, in 1979 [15]. Starting in 1982, all water bodies in Dhofar were systematically screened for presence of *B. arabica*, which revealed presence of the snail in a total of 22 water bodies out of 120, most of which were located in remote mountainous areas, and difficult to reach [17]. An inter-sectorial control strategy was consequently launched in 1983. It included snail control by mollusciciding with niclosamide (Bayluscide®); environmental modification of water bodies (embanking; building public latrines and increasing access to potable water); health education relying on different media channels including notice signs advising the population not to enter water in reason of the risk of schistosomiasis (Fig. 4); and population screening and treatment of positive cases.

While transmission appeared largely under control during the 1990s, a recrudescence in 2000–2001 triggered the adoption of a schistosomiasis elimination strategy relying on mass treatment with praziquantel. The strategy was launched by the Ministry of Health in 2003, with support from the World Health Organization. Elimination targets were set by the Ministry of Health of Oman and defined as parasitological prevalence ≤ 1% by Kato-Katz thick smear and serological prevalence ≤ 5% by Indirect Haemagglutinantion Assay (IHA), to be achieved in Dhofar Governorate by 2012 [MoH, unpublished]. Six rounds of mass treatment with praziquantel 40–60 mg/kg targeting children and adults were implemented at approximately annual intervals between April 2007 and September 2013 (treated population ranged between 4 000 and 15 000; see Table 1). Over this period, complementary public-health interventions continued to be implemented but on a lesser scale, although tilapia fish were introduced in some water bodies for biological control of snails. Health education activities consisted in renovating existing notice signs and introducing new ones, and in providing similar messages to children in school (through health education sessions) and adults in health centres (through posters, and health staff’s advice).

**Table 1:** Mass chemotherapy with praziquantel, 2007 – 2013

Round	Year	Wilayat	Target population	Population treated
1st	April 2007	Salalah	Schoolchildren	4,302
2nd	October 2007	Salalah	Schoolchildren and adults	
3rd	2008	Salalah	Schoolchildren and adults	5,148
-	2009	-	Not Implemented	0
4th	2010	Taqah	Schoolchildren and adults	1,760
5th	2011	Salalah, Taqah, Mirbat	Schoolchildren and adults	15,795
6th and final round	2013	Taqah, Mirbat	Schoolchildren and adults	4,960

## Methods

From 1982 to 2011, schistosomiasis trends were monitored through active parasitological screenings of population living in the endemic areas (both Omani and expatriates), mainly schoolchildren aged 6–17 years, an exercise that was followed by treatment of positive cases and, from 2007, by the implementation of mass chemotherapy with praziquantel in selected areas.

From 2001 onwards, yearly serological assessments were also implemented among schoolchildren (all Omani). As such, between 2001 and 2011, both stool and serological assessments were carried out. As in 2011 no individuals were found to test positive to stool examination, the Ministry of Health decided to stop parasitological screening and only rely on serological investigations.

Between 1982 and 2011, stool samples were collected by sanitary inspectors and processed at the Governorate Public Health Laboratory in Salalah, the capital city of Dhofar. The technique employed was Kato-Katz thick smear [18].

Serological assessments were conducted among schoolchildren every year between 2001 and 2014. Schools were selected from affected localities in the wilayats of Salalah, Taqah, and Mirbat. The last survey conducted prior to the commencement of mass treatment interventions was implemented in 2007; the last one in 2014, 1 year after the last distribution of praziquantel. The inclusion criteria were (i) 7^th^ grade students (12–14 years old) at the time of the survey; (ii) from schools located in endemic areas in Dhofar Governorate (i.e. from selected localities in the area suspect for active transmission). The exclusion criteria were (i) student not registered in 7th grade, (ii) unwillingness to participate or (iii) impossibility to take blood sample for any reason.

Children recruited in serological monitoring and evaluation activities were asked to provide blood samples for serological screening by IHA [19], with the aim of detecting antibodies against adult *S. mansoni* worm antigens, and thus documenting both current and past infection (elevated antibody levels are still detectable many years after cure [20]). Blood samples were collected in the field by health personnel and transported to the Governorate Public Health Laboratory in Salalah for processing.

The aim of the stool and serological screening was to monitor trends in epidemiology of schistosomiasis, and, from 2007 (baseline) onwards, to assess the impact of mass chemotherapy with praziquantel over the following years. Active surveillance by population screening was meant to complement passive surveillance implemented through the routine health services of Oman, based on the consideration that the sensitivity of the latter strategy would be too low to detect autochthonous cases of schistosomiasis (who would most likely be asymptomatic and of low intensity of infection in reason of its reduced levels of transmission).

## Results

**Fig. 5:**
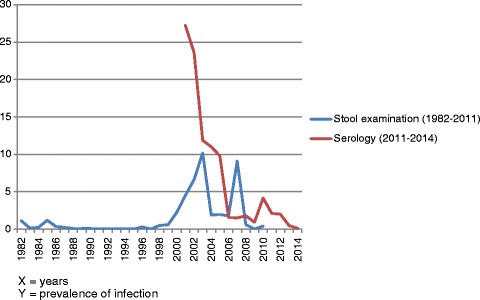
Decline of parasitological and serological prevalence of infection with *S. mansoni* in Dhofar

Table 2 and Fig. 5 show numbers screened and testing positive by type of examination, between 1982 and 2014, and the corresponding levels of parasitological and serological prevalence of infection. Overall, a decreasing trend in both parasitological prevalence and seroprevalence of *S. mansoni* infection was observed among screened individuals in Dhofar Governorate during the period under study.

**Table 2:** Number of schistosomiasis cases actively detected annually in Dhofar: 1982 – 2014

Year	Stool Examination				Serology Test	
	Number screened	Omani positive	Non-Omani positive	Total positive	Number screened	Positive (all Omani)
1982	2,038	16	7	23 (1.12%)	--	--
1983	7,278	4	6	10 (0.13%)		--
1984	3,880	4	6	10 (0.25%)	--	--
1985	3,352	16	24	40 (1.19%)	--	--
1986	5,663	10	10	20 (0.35%)	--	--
1987	3,977	3	7	10 (0.25%)	--	--
1988	5,642	2	3	5 (0.08%)	--	--
1989	2,739	2	1	3 (0.1%)	--	--
1990	2,645	3	0	3 (0.11%)	--	--
1991	1,500–2,000	0	0	0	--	--
1992	1,500–2,000	0	0	0	--	--
1993	1,500–2,000	0	0	0	--	--
1994	1,500–2,000	0	0	0	--	--
1994	1,500–2,000	0	0	0	--	--
1996	654	2	0	2 (0.3%)	--	--
1997	444	0	0	0	--	--
1998	200	1	0	1 (0.5%)	--	--
1999	168	1	0	1 (0.59%)	--	--
2000	578	13	0	13 (2.24%)	--	--
2001	800	36	0	36 (4.5%)	511	139 (27.2%)
2002	1,237	81	1	82 (6.62%)	305	72 (23.6%)
2003	796	81	0	81 (10.17%)	2 418	287 (11.86%)
2004	748	14	0	14 (1.87%)	3 745	413 (11.02%)
2005	763	15	0	15 (1.96%)	5 633	551 (9.78%)
2006	448	5	3	5 (1.78%)	2 639	42 (1.59%)
2007	165	15	0	15 (9.09%)	3 210	48 (1.49%)
Start of mass treatment with praziquantel						
2008	167	1	0	1 (0.59%)*	9 188	165 (1.79%)
2009	265	0	0	0	3 024	28 (0.92%)
2010	246	1	0	1 (0.4%)*	3 265	136 (4.16%)
2011	109	0	0	0	2 988	63 (2.10%)
2012	--	--	--	--	2 513	51 (2.02%)
2013	--	--	--	--	2 788	14 (0.50%)*
2014	--	--	--	--	3 608	4 (0.11%)*

(*) Statistically-significant reduction from 2007 values

An increase in parasitological prevalence was however observed from the early 2000s, after several years during which the number of detected cases was very small. This finding reflects the reintroduction of transmission in rural areas of Salalah wilayat, a fact confirmed by the detection of infected *B. arabica* snails in the relevant water bodies, several year after the last specimen had been found [MoH, unpublished]. The causes of such recrudescence remain unclear, although suspicions point either to a rodent reservoir, based on the consideration that *Rattus rattus* was found to be naturally infected with *S. mansoni* in Dhofar [17], or to reintroduction of transmission by travellers from neighbouring countries (e.g. Yemen was then highly endemic for *S. mansoni*). Following the adoption of a mass-treatment-based elimination strategy by the health authorities of Oman, a statistically significant decrease (*P* < 0.001) in prevalence was observed between 2007 (last stool investigation conducted prior to the start of mass treatment) and the following years (2008–2011). Over such 4-year period, only zero or one cases were detected during each survey, compared to 15 cases in 2007.

Serological trends also show a progressive decrease in prevalence. Notably, the last survey conducted prior to the start of mass chemotherapy, in 2007, indicated that out of 3 210 children serologically screened, 48 were positive (1.49%). In 2014, following the completion of six rounds of praziquantel mass chemotherapy, 3 608 children were serologically tested and only four found to be positive (0.11%). The decrease in prevalence of infection observed between 2007 and 2014 is statistically significant (*P* < 0.001).

## Discussion

A parasitological prevalence ≤ 1% by Kato-Katz and a serological prevalence ≤ 5% by IHA, in 2006 were achieved in Dhofar in 2008 and 2006, respectively. As such, as of 2008, the country is compliant with both national elimination criteria set by the Ministry of Health of Oman (although WHO rather employs the term elimination to refer to the actual interruption of transmission [2]). The irregular but progressive decrease of parasitological prevalence, and the less rapid, but steady decline of serological prevalence of *S. mansoni* infection in areas at risk in Dhofar reflect the different phases experienced by Oman in its fight against schistosomiasis: the gradual reduction of risk of acquiring new infections in the 1980s–1990s as a probable consequence of the inter-sectorial control measures applied; the recrudescence observed in the early 2000s; and finally the impact on transmission registered from 2007 onwards, as a probable consequence of the implementation of the six rounds of mass treatment conducted until 2013.

## Conclusion

Transmission of schistosomiasis has reached very low levels throughout the area at risk in Oman; considering that serological assessments detect both current and past infections, transmission might in fact have been interrupted in some or all foci already. Additional investigations are therefore required to ensure collection of evidence that would sustain the country’s claim that schistosomiasis has been eliminated, and would enable Oman to access the WHO’s formal process to verify interruption of transmission, which is currently being established [A. Garba, personal communication].

In line with other countries which have achieved low transmission of *Schistosoma* spp. infection, such investigations should be systematically implemented throughout the area at-risk, and should rely on sensitive procedures able to rule out continuing transmission. Such procedures include molecular detection of *S. mansoni* cercariae in snail intermediate hosts [20], as well as assessment of current infection in human populations, through detection of antigens to *S. mansoni* (e.g. by circulating cathodic antigen (CCA) tests) [21, 22], or through stool sedimentation coupled with miracidia hatching test [23].

Should transmission of *S. mansoni* be shown to continue in Dhofar, although at low levels, an aggressive strategy would be required, relying on mass chemotherapy with praziquantel in all remaining foci coupled with a renewed effort on complementary public health interventions such as snail control, environmental management, improved sanitation, safe water supply, and health education. In case of successful verification of the schistosomiasis-free status, post-elimination surveillance would need to be implemented with the aim of detecting any new case and preventing reintroduction of transmission in Dhofar, at least until all neighbouring endemic countries will have achieved the same goal [2]. The experience accumulated by health authorities of Oman in controlling and monitoring schistosomiasis for a period of over 30 years will certainly prove useful in either endeavor.

## Box 1 Timeline of facts and activities related to schistosomiasis in Oman

1970s/1980s – Malacological investigations define geographical areas of Oman where snail intermediate hosts of *Schistosoma* spp. can be found

1979 – First cases of *S. mansoni* infection detected in Dhofar Governorate, Oman

1982 – Systematic screening of water bodies in Dhofar Governorate for presence of *B. arabica*; active surveillance by systematic stool examination is initiated

1983 – An inter-sectorial control strategy is launched by the Ministry of Health

1990s – Incidence of infection reaches very low levels

2001 – Active surveillance by systematic serological surveys is initiated

2003 – An elimination strategy relying on mass treatment is launched following a recrudescence in transmission

2007 – The last parasitological autochthonous case is detected through passive surveillance; mass chemotherapy with praziquantel is initiated

2010 – The last parasitological autochthonous case is detected through active surveillance

2012 – Active surveillance by systematic stool examination is discontinued

2014 – Mass chemotherapy with praziquantel is discontinued

2015 – Active surveillance by systematic serological surveys is suspended while MoH is planning the implementation of surveys aimed at confirming interruption of transmission

## Additional file

Additional file 1: Multilingual abstracts in the six official working languages of the United Nations. (PDF 766 kb)

## Abbreviations

CCA: Circulating cathodic antigen
IHA: Indirect Haemagglutination Assay
MoH: Ministry of Health
WHA: World Health Assembly
WHO: World Health Organization
