# Supplementary material for: Decline in transmission of schistosomiasis mansoni in Oman
Source: Infect Dis Poverty. 2016 Dec 12;5:112. doi: 10.1186/s40249-016-0210-1 (PMC5151125; doi:10.1186/s40249-016-0210-1)
Supplement: Additional file 1: — Multilingual abstracts in the six official working languages of the United Nations. (PDF 766 kb) [file 40249_2016_210_MOESM1_ESM.pdf]

Translation of the abstract into the six official working languages of the United Nations

تراجع انتشار البلهارسيا المونسونية في عُمان

ادريس العبيداني، ومحمد شعبان، وساتيش غوجي، وسالم الكثيري، وخالد المشيخي، وأمادو غربا، وألبيس فرانسيسكو غابريلي

ملخّص:
معلومات أساسية: أُكتشفت أول حالة إصابة بداء البلهارسيا المعوية، الذي تسببه البلهارسيا المونسونية، للمرة الأولى في عُمان عام 1979م. ويشير الاتجاه إلى إصابة الانسان بالبلهارسيا الطفيلية، والبلهارسيا السيريلوجية في المنطقة الموبوءة كان خلال الفترة من 1982-2014م، وتطابق البيانات التي أعدها الجهاز الوطني للرصد والتقييم، ومعايير القضاء على البلهارسيا، التي حددتها وزارة الصحة العمانية.
الطُرُق: أُجريت اختبارات على الطفيل والمصل على السكان (ولا سيما الأطفال) الذين يعيشون في المنطقة المعرضة لخطر البلهارسيا، في ظفار، وهي المنطقة الوحيدة الموبوءة في البلاد، على مدى ثلاثين عاماً. واستخدمت في الفحوصات طريقتا كاتو- كاتز المكثفة، ومقايسة التراص الدموي غير المباشر.
النتائج: تشير البيانات إلى انخفاض تدريجي لمعدل الإصابة بمرض البلهارسيا المنسونية خلال حقبتي الثمانينيات والتسعينيات، والظهور مرة أخرى في أوائل عام 2000م، وانخفاضاً كبيراً ملحوظاً بعد تنفيذ 6 جولات من العلاج الجماعي بعقار البرازيكوانتيل في الفترة من 2007 وحتى 2013م. وكان آخر حالات الإصابة بالطفيل 0٪ (2011)، بينما كانت آخر إصابة بالسيريولوجي (2014) 0,11٪.
الخلاصة: تراجعت معدلات الإصابة بالبلهارسيا في عمان مستويات متدنية. وتحقق معيار القضاء على المرض الذي وضعته وزارة الصحة العمانية (معدل الإصابة الطفيلية ≤ 1٪، والإصابة السيرولوجية ≤ 5٪) منذ عام 2008م. وهناك حاجة لمزيد من التقصي لتحديد ما إذا كان الحد من الانتشار قد تحقق في بعض البؤر، أو جميعها، وذلك في ضوء وضع إجراء تحقق رسمي بإشراف منظمة الصحة العالمية.

Translated from English version into Arabic by Hassan Adam, through

阿曼曼氏血吸虫病传播呈下降态势

Idris Al Abaidani, Seif Al-Abri, Mahmoud Shaban, Satish L Ghugey, Salem Al Kathery, Khalid Al-Mashikhi, Amadou Garba, Albis Francesco Gabrielli

摘要
引言：1979 年，阿曼首次报道了由感染曼氏血吸虫引起肠道血吸虫病。本研究描述了 1982-2014 年间，阿曼流行区人体感染曼氏血吸虫的寄生虫学和血清学流行趋势，数据源自国家监测和评价系统，以及以阿曼卫生部制定的血吸虫病消除标准作为评价标准。
方法：1982-2014 年，对居住在该国唯一流行省 Dhofar 的血吸虫病风险地区的人群（主要是儿童）进行寄生虫和血清学检测，使用 Kato-Katz 厚涂片和间接血凝测定法。
结果：数据表明，整个 20 世纪 80 至 90 年代，曼氏血吸虫发病率逐年下降；21 世纪早期出现回升；2007 年至 2013 年间实施六轮大规模吡喹酮治疗后，发病率显著降低。最新寄生虫学患病率为 0%（2011 年），血清学阳性率为 0.11%（2014）。
结论：阿曼血吸虫病传播已达到很低的水平。自 2008 年以来，阿曼血吸虫病患病率已符合卫生部制定的消除标准（寄生虫学患病率≤1%，血清学阳性率≤5%）。鉴于世卫组织主持建立了正式的核查程序，需要进一步调查以评估在某些或所有地区是否已实现传播阻断。

Translated from English version into Chinese by Jin Chen, edited by Pin Yang

Diminution du taux de transmission de la schistosomiase due au Schistosomia mansoni à Oman

Idris Al Abaidani, Seif Al-Abri, Mahmoud Shaban, Satish L Ghugey, Salem Al Kathery, Khalid Al-Mashikhi, Amadou Garba, Albis Francesco Gabrielli

Résumé
Contexte: la schistosomiase intestinale due au Schistosomia mansoni a été signalée pour la première fois à Oman en 1979. Nous nous attachons à décrire la tendance en termes de prévalence parasitologique et sérologique de l'infection humaine par le S. mansoni dans sa région endémique de 1982 à 2014 et la conformité des données générées par le système national de suivi et d'évaluation par rapport aux critères d'élimination de la schistosomiase définis par le Ministère de la Santé d'Oman.
Méthodes: les évaluations parasitologiques et sérologiques ont été réalisées sur une population (principalement infantile) vivant dans la région à risque de contraction de la schistosomiase à Dhofar, le seul gouvernorat endémique du pays, pendant une période de plus de 30 ans. La mesure sur goutte épaisse de Kato-Katz et le test d'hémagglutination indirecte ont à cette occasion été utilisés.
Résultats: les données indiquent une diminution progressive de la prévalence du S. mansoni tout au long des années 1980 et 1990, une recrudescence au début des années 2000, puis une baisse plus marquée à la suite de la mise en œuvre de six séries de traitement de masse au praziquantel de 2007 à 2013. Le dernier taux de prévalence parasitologique (2011) atteignait 0 %, tandis que le dernier taux de prévalence sérologique (2014) s'élevait à 0,11 %.
Conclusions: la transmission de la schistosomiase a atteint des taux très faibles à Oman. Les critères d'élimination établis par le Ministère de la Santé d'Oman (prévalence parasitologique < 1 % et prévalence sérologique < 5 %) sont satisfaits depuis 2008. De plus amples examens sont requis afin d'évaluer si l'interruption de la transmission a été obtenue dans certains ou tous les foyers afin d'établir un processus de vérification formelle sous l'égide de l'OMS.

Translated from English version into French by eric ragu, through

TRANSLATORS
WITHOUT BORDERS

Снижение заболеваемости и передачи шистосомоза (*Schistosoma mansoni*) в Омане

Idris Al Abaidani, Seif Al-Abri, Mahmoud Shaban, Satish L Ghugey, Salem Al Kathery, Khalid Al-Mashikhi, Amadou Garba, Albis Francesco Gabrielli

Аннотация
Актуальность исследования: Кишечный шистосомоз, вызываемый *Schistosoma mansoni*, был впервые отмечен в Омане в 1979 г. В статье описаны тенденции изменения паразитологической и серологической распространенности инфицирования человека вирусом *S. mansoni* в эндемичных районах за период 1982-2014, а также соответствие данных, полученных от национальной системы мониторинга и оценки, с критериями ликвидации шистосомоза, установленными Министерством здравоохранения Омана.
Методы исследования: Паразитологические и серологические оценки, с использованием методов Като-Катца и непрямой гемагглютинации (Indirect Haemagglutination Assay), проводились в течение более 30 лет среди населения (в основном детского), проживающего в зоне риска заболевания шистосомозом в Дофаре, единственной эндемичной Губернии страны.
Результаты исследования: Данные показывают постепенное снижение распространенности *S. mansoni* на протяжении 1980-х и 1990-х, вспышку заболевания в начале 2000-х годов, а также более выраженное снижение распространенности после проведения шести раундов массового лечения празиквантелом в период с 2007 по 2013 г. Последние данные показывают, что паразитологическая распространенность (2011) составила 0%, в то время как серологическая распространенность (2014) составила 0,11%.
Заключение: Передача шистосомоза в Омане достигла очень низкого уровня. Критерии для ликвидации, установленные Министерством здравоохранения Омана (паразитологическая распространенность ≤ 1% и серологическая распространенность ≤ 5%), выполняются, начиная с 2008 года. Для начала формального, проводимого под эгидой ВОЗ, процесса подтверждения прерывания передачи заболевания, необходимы дальнейшие исследования, чтобы оценить, было ли достигнуто прерывание передачи только в некоторых или же во всех очагах распространения.

Translated from English version into Russian by Alexei Mikhailov

Disminución de la transmisión de esquistosomiasis mansoni en Omán

Idris Al Abaidani, Seif Al-Abri, Mahmoud Shaban, Satish L Ghugey, Salem Al Kathery, Khalid Al-Mashikhi, Amadou Garba, Albis Francesco Gabrielli

Resumen

Antecedentes: La esquistosomiasis intestinal provocada por *Schistosoma mansoni* fue observada por primera vez en Omán, en 1979. Describimos la tendencia de la prevalencia parasitológica y serológica de la infección en humanos con *S. mansoni* en su área endémica durante el periodo 1982-2014, y el cumplimiento de los datos generados por el sistema nacional de seguimiento y evaluación con los criterios de eliminación de la esquistosomiasis establecidos por el Ministerio de Sanidad de Omán.
Métodos: Durante un periodo de 30 años, se realizaron evaluaciones parasitológicas y serológicas en la población (principalmente niños) que habita en la zona de riesgo para la esquistosomiasis de Dhofar, la única gobernación del país donde es endémica. Las técnicas empleadas fueron la técnica de frotis grueso de Kato-Katz y la hemaglutinación indirecta.
Resultados: Los datos muestran una reducción progresiva de la prevalencia de *S. mansoni* a lo largo de las décadas de los 80 y los 90, un agravamiento a comienzos de la década de 2000, y un descenso más acusado tras la aplicación de seis tandas de tratamiento masivo con praziquantel desde 2007 hasta 2013. La última prevalencia parasitológica (2011) fue del 0% mientras que la última prevalencia serológica (2014) fue del 0,11%.
Conclusiones: La transmisión de la esquistosomiasis ha llegado a niveles muy bajos en Omán. Desde 2008 se han cumplido los criterios de eliminación establecidos por el Ministerio de Sanidad de Omán (prevalencia parasitológica $\leq$ 1% y prevalencia serológica $\leq$ 5%). Es necesario realizar investigaciones adicionales para evaluar si la interrupción de la transmisión se ha logrado en todos los focos o solo en algunos, con vistas a establecer una proceso de verificación formal bajo los auspicios de la OMS.

Translated from English version into Spanish by Barbara Gutierrez Teira, through

TRANSLATORS
WITHOUT BORDERS
